# Diagnostic Performance of ANCA IIF in Relation to PR3 and MPO Antibodies: Impact of Formalin Reactivity in a Large Real-World Cohort

**DOI:** 10.3390/diagnostics16070996

**Published:** 2026-03-26

**Authors:** Baris Can, Arzu Aksit Ilki

**Affiliations:** Department of Medical Microbiology, Marmara University School of Medicine, Istanbul 34854, Turkey; ailki@marmara.edu.tr

**Keywords:** ANCA, PR3, MPO, indirect immunofluorescence, formalin fixation, diagnostic concordance, ANCA-associated vasculitis

## Abstract

**Background**: The diagnostic interpretation of antineutrophil cytoplasmic antibody (ANCA) testing remains challenging due to variable concordance between indirect immunofluorescence (IIF) patterns and antigen-specific assays targeting proteinase 3 (PR3) and myeloperoxidase (MPO). The additional value of formalin fixation in distinguishing true perinuclear ANCA (P-ANCA) patterns from redistribution artifacts in routine laboratory practice remains incompletely defined. **Methods**: We conducted a retrospective real-world analysis of 7276 patients who underwent concurrent ANCA IIF (ethanol- and formalin-fixed substrates), anti-PR3, and anti-MPO testing between 2022 and 2024. Concordance between IIF and antigen-specific assays was assessed using cross-tabulation and Cohen’s kappa statistics. Associations were evaluated using chi-square tests and odds ratios (ORs) with 95% confidence intervals (CIs). The impact of formalin reactivity within P-ANCA patterns and the relationship between ANCA titer strength and antigen positivity were analyzed using the chi-square test for trend. **Results**: Concordance between ANCA IIF and PR3 antibodies was poor (κ = 0.109; *p* < 0.001), with 59% of PR3-positive patients being IIF-negative. Agreement between ANCA IIF and MPO antibodies was stronger but remained modest (κ = 0.221; *p* < 0.001); 77% of MPO-positive patients were IIF-positive. Formalin-resistant P-ANCA patterns showed a strong association with MPO positivity (OR = 67.3; 95% CI 33.3–136.6; *p* < 0.001), whereas MPO positivity was uncommon in formalin-sensitive patterns. Increasing ANCA titers were associated with progressively higher MPO positivity (chi-square test for trend, *p* < 0.001), rising from 0.4% in ANCA-negative cases to 33.3% in the highest observed titer category. Dual PR3/MPO positivity was rare (0.16%). **Conclusions**: In this large cohort, ANCA IIF demonstrated differential diagnostic performance depending on antibody specificity. IIF showed limited sensitivity for PR3-associated autoimmunity, whereas MPO positivity correlated more strongly with IIF patterns and formalin resistance. Integration of antigen-specific assays with formalin-based pattern interpretation may improve the interpretative accuracy of ANCA testing and should be considered in routine laboratory algorithms.

## 1. Introduction

Antineutrophil cytoplasmic antibodies (ANCAs) are central serological markers in the diagnosis of ANCA-associated vasculitis (AAV), a group of necrotizing small-vessel vasculitides that include granulomatosis with polyangiitis and microscopic polyangiitis [1,2,3]. AAV represents a rare but potentially life-threatening autoimmune disease spectrum associated with significant morbidity and mortality if not promptly recognized and treated [4,5]. The classification and diagnostic framework of AAV has evolved considerably over the past decade, culminating in the revised international consensus on ANCA testing and the 2022 ACR/EULAR classification criteria [1,2,3].

Traditionally, ANCA detection has relied on indirect immunofluorescence (IIF) performed on ethanol-fixed neutrophils, allowing the identification of cytoplasmic (C-ANCA) and perinuclear (P-ANCA) staining patterns [4,6]. However, advances in immunoassay technology have enabled the development of antigen-specific assays targeting proteinase 3 (PR3) and myeloperoxidase (MPO), which are now widely incorporated into contemporary diagnostic algorithms [1,7,8]. Large multicenter evaluations and meta-analyses have demonstrated that high-quality antigen-specific immunoassays may provide superior specificity compared with IIF, although inter-assay and inter-platform variability persists [4,7,9].

Despite international consensus recommendations favoring antigen-specific immunoassays as primary screening tools [1,6], IIF remains routinely used in many laboratories worldwide due to historical practice patterns, compatibility with automated image analysis systems, and cost considerations [6,10]. Importantly, concordance between IIF patterns and PR3/MPO antibody positivity is not absolute. Several studies have reported discordant results, with antigen-positive patients lacking classical fluorescence patterns and, conversely, fluorescence-positive samples without corresponding antigen specificity [4,7,11]. These discrepancies raise ongoing questions regarding the standalone diagnostic utility of IIF and the optimal integration of testing modalities within real-world laboratory workflows.

Fixation methods further complicate fluorescence interpretation. Ethanol fixation may induce the redistribution of cytoplasmic antigens toward the nucleus, thereby generating artifactual perinuclear staining patterns [12,13]. Formalin fixation has been proposed as a complementary strategy to differentiate true MPO-associated P-ANCA from ethanol-induced redistribution phenomena [12,13]. Although dual-fixed neutrophil substrates have demonstrated improved interpretative accuracy in controlled methodological studies [13], the real-world diagnostic contribution of formalin reactivity in large routine laboratory cohorts remains insufficiently characterized.

Beyond qualitative pattern recognition, semi-quantitative ANCA titers may provide additional diagnostic and potentially prognostic information. Previous investigations have suggested that antibody specificity, subtype, and titer strength may correlate differently with disease phenotype, relapse risk, and clinical course [14,15,16,17,18]. However, the relationship between fluorescence intensity categories and concurrent antigen-specific PR3 and MPO positivity has not been consistently evaluated in large, unselected laboratory populations.

In this context, the primary objective of the present study was to evaluate the concordance between ANCA indirect immunofluorescence (IIF) patterns and antigen-specific PR3 and MPO assays in a large real-world laboratory cohort.

Secondary objectives included assessing the diagnostic impact of formalin reactivity in P-ANCA patterns and investigating the relationship between ANCA titer strength and antigen-specific antibody positivity.

By analyzing these parameters together, we aimed to better characterize the real-world diagnostic performance of ANCA IIF in comparison with antigen-specific assays used in routine clinical laboratories.

## 2. Materials and Methods

### 2.1. Study Design and Population

This retrospective single-center study was conducted by the Department of Medical Microbiology and included consecutive patients from Marmara University Pendik Training and Research Hospital who underwent concurrent ANCA indirect immunofluorescence (IIF), anti-PR3, and anti-MPO testing between January 2022 and December 2024. Only patients with complete results for all three assays were included in the final analysis. To avoid duplication bias, only the first available test result per patient during the study period was considered.

Demographic data (age and sex), ANCA IIF pattern, formalin reactivity status, and antigen-specific antibody results were extracted from the laboratory information system.

The study was conducted in accordance with the Declaration of Helsinki and approved by the Marmara University Faculty of Medicine Non-Interventional Clinical Research Ethics Committee (Approval No. 09.2026.26-0229; approved on 20 February 2026).

### 2.2. ANCA Indirect Immunofluorescence

ANCA testing was performed using commercially available indirect immunofluorescence kits (EUROIMMUN, Lübeck, Germany) according to the manufacturer’s instructions. Substrates included ethanol-fixed human granulocytes, formalin-fixed granulocytes, and HEp-2 cells.

Serum samples were screened at an initial dilution of 1:10. Fluorescence patterns were evaluated using an automated image acquisition system (EUROPattern Analyzer; EUROIMMUN, Lübeck, Germany) and subsequently interpreted by experienced laboratory specialists. Final pattern classification (C-ANCA, P-ANCA, atypical ANCA, or negative) was confirmed by a laboratory medicine specialist to ensure diagnostic consistency.

Fluorescence interpretation was performed according to the routine diagnostic workflow of the laboratory by experienced laboratory specialists. Because the analysis was retrospective and based on routinely generated laboratory data, observers were not blinded to the overall laboratory workflow results. Formal inter-observer agreement analysis was not performed; however, final pattern classification was reviewed and confirmed by a laboratory medicine specialist to maintain diagnostic consistency.

ANCA titers were semi-quantitatively categorized according to fluorescence intensity and dilution levels as follows: weak positive (1:10), + (>1:10 to <1:32), ++ (≥1:32 to <1:100), +++ (≥1:100 to <1:320), and ++++ (≥1:320).

For statistical purposes, titers were converted into an ordinal score ranging from 0 to 4, where 0 represented negative results, 1 represented weak positivity (1:10), 2 corresponded to + intensity, 3 corresponded to ++ intensity, and 4 corresponded to +++ intensity. No samples reached the ++++ (≥1:320) category during the study period. Therefore, score 4 in the ordinal model represented the highest observed fluorescence intensity category (+++; ≥1:100 to <1:320).

Formalin reactivity in P-ANCA patterns was classified as formalin-sensitive or formalin-resistant based on the persistence of fluorescence following formalin fixation.

### 2.3. Anti-PR3 and Anti-MPO Assays

Anti-PR3 and anti-MPO antibodies were measured using commercially available enzyme-linked immunosorbent assay (ELISA) kits (EUROIMMUN, Lübeck, Germany) according to the manufacturer’s instructions. Results were reported in relative units per milliliter (RU/mL).

A cut-off value of 20 RU/mL was applied for both assays in accordance with the manufacturer’s recommendations. Values <20 RU/mL were considered negative, whereas values ≥20 RU/mL were considered positive. For concordance analyses, antibody results were dichotomized into binary categories (positive/negative).

The EUROIMMUN platform was selected because it provides standardized IIF substrates and antigen-specific ELISA assays for PR3 and MPO, which are widely used in routine clinical laboratories. Using assays from the same manufacturer ensured methodological consistency and enabled direct comparison between fluorescence patterns and antigen-specific immunoassays within a single analytical platform.

### 2.4. Statistical Analysis

Categorical variables were expressed as numbers and percentages. Continuous variables were presented as median and interquartile range (IQR). Concordance between ANCA IIF and the antigen-specific assays was assessed using cross-tabulation and Cohen’s kappa coefficient.

Associations between formalin reactivity and MPO positivity were evaluated using contingency tables and reported as odds ratios (ORs) with 95% confidence intervals (CIs). Trends in PR3 and MPO positivity across ANCA titer categories were analyzed using the chi-square test for trend.

All statistical analyses were performed using Python (version 3.10; Python Software Foundation, Wilmington, DE, USA). A two-sided *p*-value < 0.05 was considered statistically significant.

## 3. Results

### 3.1. Study Population

**Figure 1:**
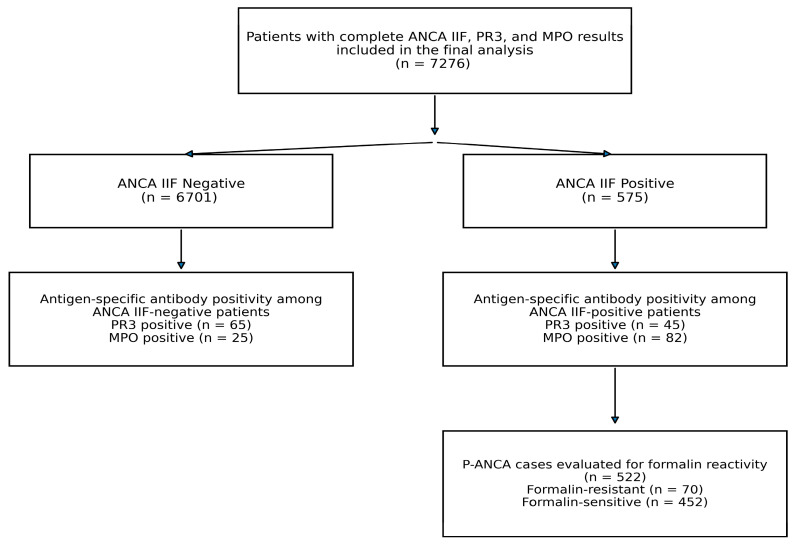
Study flow diagram of patient inclusion and distribution of ANCA IIF results, antigen-specific antibody positivity, and formalin reactivity among the P-ANCA cases.

A total of 7276 patients were included in the final analysis. The selection of the study population and the distribution of ANCA IIF and antigen-specific antibody results are summarized in Figure 1.

The demographic and serological characteristics of the study population are presented in Table 1.

### 3.2. Concordance Between ANCA IIF and PR3 Antibodies

PR3 positivity was identified in 110 patients (1.5% of the total cohort). The distribution of PR3 results according to ANCA IIF status is presented in Table 2.

PR3 positivity was observed in 7.8% of ANCA IIF-positive patients compared with 1.0% of ANCA IIF-negative patients. Notably, 59% of PR3-positive cases were ANCA IIF-negative, indicating limited concordance and reduced sensitivity of IIF for PR3-associated serology.

### 3.3. Concordance Between ANCA IIF and MPO Antibodies

MPO positivity was identified in 107 patients (1.5% of the total cohort). The distribution of MPO results according to ANCA IIF status is presented in Table 3.

MPO positivity was observed in 14.3% of ANCA IIF-positive patients compared with 0.4% of ANCA IIF-negative patients. Notably, 77% of MPO-positive cases were ANCA IIF-positive, demonstrating stronger concordance compared with PR3, although overall agreement remained modest.

### 3.4. Impact of Formalin Reactivity in P-ANCA Patterns

The association between formalin reactivity and MPO positivity among P-ANCA cases is presented in Table 4.

Formalin-resistant P-ANCA patterns demonstrated a markedly higher rate of MPO positivity compared with formalin-sensitive patterns (75.7% vs. 4.4%). The magnitude of association (OR = 67.3; 95% CI 33.3–136.6; *p* < 0.001) indicates a substantial discriminatory effect of formalin resistance in identifying MPO-associated P-ANCA and supports its practical utility in routine laboratory interpretation.

### 3.5. ANCA Titer Strength and Antigen Positivity

**Figure 2:**
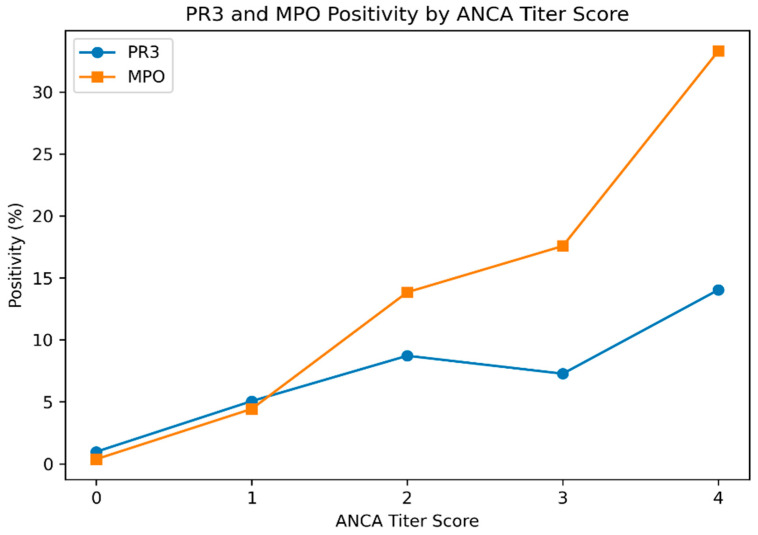
PR3 and MPO positivity rates according to ANCA titer score. MPO demonstrates a marked titer-dependent increase in positivity compared with PR3.

The relationship between ANCA titer strength and antigen-specific antibody positivity is presented in Table 5 and illustrated in Figure 2.

MPO positivity demonstrated a clear and progressive titer-dependent increase, rising from 0.4% in ANCA-negative cases to 33.3% in the highest titer category. In contrast, PR3 positivity showed a weaker and less linear association with increasing ANCA titer (Figure 2).

### 3.6. Dual PR3 and MPO Antibody Positivity

Simultaneous positivity for both PR3 and MPO antibodies was identified in 12 patients, corresponding to 0.16% of the total cohort. This finding confirms that dual PR3/MPO antibody positivity is uncommon in routine diagnostic practice.

We additionally evaluated combined antigen-specific antibody positivity defined as positivity for either PR3 or MPO antibodies. The distribution of combined PR3/MPO positivity according to ANCA IIF status is presented in Table 6.

Combined antigen positivity was more frequently observed among the ANCA IIF-positive patients compared with the ANCA IIF-negative patients. However, a substantial proportion of antigen-positive cases remained ANCA IIF-negative, indicating incomplete concordance between fluorescence-based screening and antigen-specific immunoassays.

## 4. Discussion

In this large real-world laboratory cohort comprising 7276 patients undergoing concurrent ANCA IIF, anti-PR3, and anti-MPO testing, we identified differential concordance patterns between indirect immunofluorescence and antigen-specific immunoassays. The principal findings can be summarized as follows: (1) concordance between ANCA IIF and PR3 antibodies was poor; (2) agreement between ANCA IIF and MPO antibodies was significantly stronger, although still modest; (3) formalin-resistant P-ANCA patterns were strongly associated with MPO positivity; and (4) increasing ANCA titers were associated with progressively higher MPO positivity rates.

Our data demonstrate that PR3 positivity frequently occurs in ANCA IIF-negative patients. Nearly 60% of PR3-positive cases were IIF-negative, and the kappa coefficient indicated only slight agreement between the two testing modalities. In general, kappa values below 0.20 are interpreted as reflecting slight agreement, whereas values between 0.21 and 0.40 represent fair agreement. Accordingly, the observed κ value for PR3 indicates limited concordance between fluorescence-based pattern recognition and antigen-specific serology in routine diagnostic settings. These findings are consistent with prior studies suggesting that PR3-ANCA-associated disease may exhibit weaker or even absent classical fluorescence patterns compared with MPO-associated disease [19,20,21,22]. Differences in antigen accessibility, epitope conformation, and assay platform variability have been proposed as potential explanations for this phenomenon [20,21,22]. In contrast, MPO antibodies showed better concordance with ANCA IIF in our cohort, reinforcing previous observations that MPO-associated autoimmunity more consistently produces classical perinuclear fluorescence patterns [19,23]. Collectively, these findings highlight that the diagnostic performance of IIF is intrinsically influenced by antibody specificity and should not be interpreted uniformly across PR3- and MPO-associated autoimmunity.

One of the most clinically relevant observations in this study is the diagnostic impact of formalin reactivity in P-ANCA patterns. Formalin-resistant P-ANCA cases demonstrated a markedly higher rate of MPO positivity, whereas MPO positivity was uncommon in formalin-sensitive patterns. This association is biologically plausible, as ethanol fixation promotes the redistribution of cytoplasmic antigens toward the nucleus, potentially generating artifactual perinuclear staining, whereas formalin fixation preserves cytoplasmic localization [12,13,14,24]. Methodological investigations have previously shown improved specificity when dual-fixed neutrophil substrates are employed [13]; however, large-scale validation in routine laboratory populations has remained limited. Our findings provide real-world confirmation that formalin resistance represents a robust and clinically meaningful discriminator of true MPO-associated serology. However, the number of formalin-resistant P-ANCA cases in our cohort was relatively limited, which should be taken into account when interpreting the magnitude of this association. In practical terms, the persistence of P-ANCA fluorescence after formalin fixation may serve as an additional interpretative indicator supporting MPO-associated autoimmunity.

We further observed a clear titer-dependent increase in MPO positivity. MPO positivity rose from 0.4% in ANCA-negative cases to 33.3% in patients within the highest observed titer category (score 4 in the ordinal model, corresponding to +++ intensity; ≥1:100 to <1:320). This pattern aligns with previous studies indicating that antibody titers may correlate with disease phenotype, relapse risk, or immunologic burden, particularly in MPO-associated vasculitis [15,16,17,18,25,26]. In contrast, PR3 positivity demonstrated a weaker and less linear relationship with fluorescence intensity, supporting the concept that PR3- and MPO-ANCA represent biologically distinct disease subsets rather than interchangeable serological markers [19,27]. These differences may reflect underlying immunopathogenic heterogeneity between PR3- and MPO-driven autoimmunity.

The low rate of dual PR3/MPO positivity in our cohort is consistent with previously reported frequencies in large clinical series [28,29]. Although dual seropositivity has been described and may be associated with specific clinical phenotypes or transitional disease states [28], it remains uncommon in routine diagnostic practice. Accordingly, simultaneous PR3 and MPO positivity should prompt careful clinical correlation rather than be interpreted as a typical serologic presentation.

From a clinical and laboratory perspective, our findings support current international recommendations emphasizing the central role of antigen-specific immunoassays within diagnostic algorithms for suspected AAV [1,6]. While high-quality immunoassays demonstrate strong performance characteristics [7,9], IIF continues to provide complementary interpretative value, particularly when formalin reactivity is incorporated into pattern assessment. An integrated testing strategy combining antigen specificity with fixation-based pattern discrimination may therefore offer optimal diagnostic resolution in routine practice [1,4,9]. In practical terms, laboratories that continue to use ANCA IIF may benefit from incorporating formalin-fixed substrates into their routine interpretative algorithms, particularly when evaluating P-ANCA patterns in patients with suspected MPO-associated vasculitis.

This study has several limitations. First, clinical outcome data and longitudinal follow-up were not available, precluding correlation with confirmed vasculitis diagnoses, disease activity indices, organ involvement, or relapse events. Serial antibody dynamics and their prognostic implications, which have been explored in longitudinal cohorts [15,16,25], could therefore not be evaluated in the present analysis.

Consequently, the present study cannot determine the diagnostic sensitivity or specificity of the evaluated assays for confirmed ANCA-associated vasculitis. Instead, the results primarily reflect the analytical and interpretative relationships between IIF patterns and antigen-specific assays in routine laboratory practice.

Second, only patients with complete ANCA IIF, PR3, and MPO results were included in the analysis. This inclusion criterion may introduce selection bias, as clinicians may preferentially request antigen-specific testing in patients with higher suspicion of autoimmune disease.

Third, this was a single-center retrospective study, which may limit generalizability to other laboratory settings. Nevertheless, the large sample size, standardized testing platform, and real-world laboratory setting enhance the robustness and applicability of our findings. Future prospective studies integrating clinical outcomes and longitudinal follow-up will be important to further clarify the diagnostic and prognostic implications of different ANCA testing strategies.

## 5. Conclusions

In this large real-world cohort, concordance between ANCA IIF and antigen-specific assays differed substantially according to antibody specificity. Agreement between IIF and PR3 antibodies was poor, whereas MPO antibodies demonstrated relatively stronger—yet still limited—concordance with fluorescence patterns. Formalin-resistant P-ANCA patterns were strongly associated with MPO positivity, underscoring the diagnostic value of dual fixation in routine laboratory interpretation. Moreover, increasing ANCA titers were associated with progressively higher MPO positivity, indicating a clear titer-dependent relationship.

These findings suggest that ANCA IIF should not be used as a standalone screening tool, particularly in suspected PR3-associated disease, and that the integration of antigen-specific immunoassays with formalin-based pattern discrimination may improve the interpretative accuracy of ANCA testing and support laboratory decision-making in patients with suspected ANCA-associated vasculitis.

## Figures and Tables

**Table 1:** Demographic and serological characteristics of the study cohort (*n* = 7276).

Age, Years *	Male, *n* (%)	Female, *n* (%)	ANCA IIF Positive, *n* (%)	PR3 Positive, *n* (%)	MPO Positive, *n* (%)
50 (39–61)	2725 (37.4)	4551 (62.6)	575 (7.9)	110 (1.5)	107 (1.5)

* Data are presented as median (interquartile range) for continuous variables and number (percentage) for categorical variables. IIF, indirect immunofluorescence; PR3, proteinase 3; MPO, myeloperoxidase.

**Table 2:** Concordance between ANCA IIF and anti-PR3 antibodies (*n* = 7276).

	PR3 Negative, *n*	PR3 Positive, *n*	Total, *n*
ANCA IIF Negative	6636	65	6701
ANCA IIF Positive	530	45	575
Total	7166	110	7276

Agreement statistics: Cohen’s κ = 0.109; chi-square test, *p* < 0.001. PR3, proteinase 3; IIF, indirect immunofluorescence.

**Table 3:** Concordance between ANCA IIF and anti-MPO antibodies (*n* = 7276).

	MPO Negative, *n*	MPO Positive, *n*	Total, *n*
ANCA IIF Negative	6676	25	6701
ANCA IIF Positive	493	82	575
Total	7169	107	7276

Agreement statistics: Cohen’s κ = 0.221; chi-square test, *p* < 0.001. MPO, myeloperoxidase; IIF, indirect immunofluorescence.

**Table 4:** Association between formalin reactivity and MPO positivity among P-ANCA cases.

Formalin Reactivity	MPO Negative, *n*	MPO Positive, *n*	Total, *n*
Resistant	17	53	70
Sensitive	432	20	452

MPO positivity rate: 75.7% in formalin-resistant cases vs. 4.4% in formalin-sensitive cases. Odds ratio (OR) = 67.3; 95% confidence interval (CI): 33.3–136.6; chi-square test, *p* < 0.001. MPO, myeloperoxidase.

**Table 5:** PR3 and MPO positivity according to ANCA titer score.

ANCA Titer Score	*n*	PR3 Positive, %	MPO Positive, %
0 (Negative)	6701	1.0	0.4
1 (Weak)	158	5.1	4.4
2 (+)	195	8.7	13.9
3 (++)	165	7.3	17.6
4 (+++)	57	14.0	33.3

Titer categories are defined as follows: + (>1:10 to <1:32), ++ (≥1:32 to <1:100), +++ (≥1:100 to <1:320). Trend analysis: chi-square test for trend, MPO *p* < 0.001; PR3 *p* = 0.002.

**Table 6:** Concordance between ANCA IIF and combined PR3/MPO antibody positivity (*n* = 7276).

	PR3/MPO Negative, *n*	PR3/MPO Positive, *n*	Total, *n*
ANCA IIF Negative	6548	153	6701
ANCA IIF Positive	523	52	575
Total	7071	205	7276

PR3/MPO positivity was defined as positivity for at least one of the two antibodies (PR3 or MPO).

## Data Availability

The data presented in this study are available from the corresponding author upon reasonable request. The data are not publicly available due to institutional and privacy restrictions.
